# First report of *Sarcocystis* spp. (Apicomplexa, Sarcocystidae) in *Lagostomus maximus* (Desmarest, 1917) (Rodentia, Chinchillidae) in Argentina

**DOI:** 10.1016/j.ijppaw.2023.03.001

**Published:** 2023-03-06

**Authors:** Victoria Canova, Elisa Helman, María del Rosario Robles, Agustín M. Abba, Gastón Moré

**Affiliations:** a Consejo Nacional de Investigaciones Científicas y Técnicas (CONICET), Godoy Cruz 2290, (C1425FQB) CABA, Buenos Aires, Argentina; b Centro de Estudios Parasitológicos y de Vectores (CEPAVE), Bv 120 e/ 60 y 64, (1900), CCT- CONICET- La Plata, Universidad Nacional de La Plata, La Plata, Buenos Aires, Argentina; c Laboratorio de Inmunoparasitología (LAINPA), Facultad de Ciencias Veterinarias, Universidad Nacional de La Plata (FCV-UNLP), La Plata (1900), Argentina; d Institute of Parasitology, Vetsuisse Faculty, University of Berne, Langgasssstrasse 122 (3012), Berne, Switzerland

**Keywords:** *Sarcocystis* spp., Plains viscacha, PCR, Argentina

## Abstract

*Sarcocystis* is a genus of intracellular parasitic protozoa that infects various species of mammals, birds, and reptiles worldwide. At least 46 *Sarcocystis* species naturally infect rodents as intermediate hosts producing tissue cysts. This study aimed to provide the first report and molecular characterisation of *Sarcocystis* spp. in muscles from plains viscacha (*Lagostomus maximus*) in Argentina. Muscle samples of 53 plains viscachas from three provinces of Argentina were processed by homogenisation and optical microscopy to detect tissue cysts. Positive samples were analysed by PCR-sequencing, using the following markers: *18S rRNA, ITS1*, and *coxI.* The *18S rRNA* and *coxI* consensus sequences were aligned with other sequences from *Sarcocystis* spp., and phylogenetic trees were constructed. Of all animals processed, 13.2% (7/53) harboured *Sarcocystis* sp. cysts. *18S rRNA* consensus sequences were obtained from four muscle samples and one individual cyst, and they showed 99.88–100% similarity, except for the cyst sequence, which showed 97.11% homology. Similarities of only 96–97% were recorded in the *18S rRNA* fragment with other *Sarcocystis* spp. whose sequences are available in the GenBank. The five *coxI* fragment sequences obtained were 100% identical and showed an identity of 99.41–99.48% with *S. canis*. For *ITS1* only short and low-quality sequences were obtained. In the phylogenetic trees, all the sequences from plains viscachas were positioned together in a branch separated from other *Sarcocystis* spp. These results could be related to new *Sarcocystis* spp. producing sarcocysts in plains viscachas. Besides, comprehensive cyst morphological analysis using TEM from the new *Sarcocystis* species will allow a description of the cyst wall ultrastructure. In this sense, further studies are needed to deepen these findings and elucidate other potential intermediate and possible definitive hosts.

## Highlights

• Muscles of 53 plains viscachas (*Lagostomus maximus)* from Argentina were collected.
• Sarcocysts were observed in seven animals, *18S rRNA* and *cox1* sequences were obtained.
• Phylogenetically the sequences were related with *S. canis*, *S. nesbitti* and a *Sarcocystis* sp.
• The *Sarcocystis* spp. in *L. maximus* from Argentina are potentially new species.

## Graphical abstract

**Figure:**
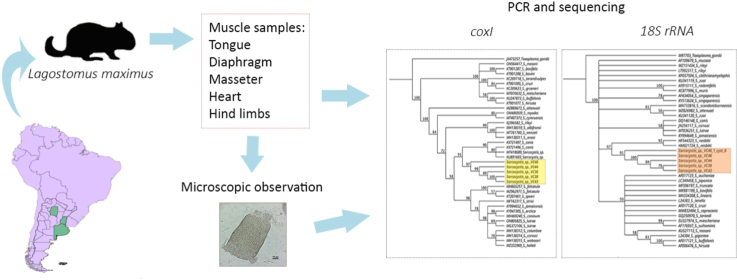

## 1 Introduction

*Sarcocystis* spp. are intracellular protozoan parasites belonging to the cyst-forming coccidia, closely related to other apicomplexans such as *Besnoitia* spp., *Neospora caninum* and *Toxoplasma gondii* (Dubey et al., 2016)*.* Most species have a predator-prey cycle producing sarcocysts in the muscles of intermediate hosts (IH) or prey and oocysts/sporocysts in the intestinal mucosa of definitive hosts (DH) carnivores and omnivores. About 200 species of this genus have been reported, infecting mammals, birds, and reptiles worldwide (Dubey et al., 2016). Humans can be hosts of some species, both as DH (*S. hominis* and *S. heydorni* in muscles from cattle and *S. suihominis* in muscles of swine) or IH (*S. nesbitti* with snakes as definitive host) (Fayer et al., 2015; Dubey et al., 2016; Rosenthal, 2021).

On the other hand, at least 46 *Sarcocystis* species used rodents as natural IH, including members of Muridae, Cricetidae, Echimyidae, Caviidae, Erethizontidae, and Sciuridae families and involving snakes, raptors, and carnivorous mammals as DH (Dubey et al., 2016; Prakas et al., 2019; Rudaitytė-Lukošienė et al., 2022).

The plains viscacha, *Lagostomus maximus* (Desmarest, 1917) (Rodentia, Chinchillidae), is endemic in South America and inhabits southeastern Bolivia, western Paraguay, and northern, eastern, and central Argentina. This rodent species, herbivorous, fossorial, and with nocturnal habits, forms colonies of up to several dozen individuals (Jackson et al., 1996; Spotorno and Patton, 2015). The plains viscacha presents economic importance since it damages fields for the construction of its underground burrows (“*vizcachera*”), and it is also used as bushmeat (Jackson et al., 1996; Spotorno and Patton, 2015). Within Chinchillidae family, the presence of *Sarcocystis microti* in *Chinchilla laniger* and infections attributable to *S. canis* in *Chinchilla* sp. were reported (Rakich et al., 1992; Dubey et al., 2016). In addition, the presence of tissue cysts in various internal organs of *L. maximus,* presumably from a *Besnoitia* sp., has been reported in Argentina (Cwirenbaum et al., 2021).

Different methods have been applied for the diagnosis of sarcocystosis, with marked differences in sensitivity and specificity (Moré et al., 2011; Dubey et al., 2016; Juozaitytė-Ngugu et al., 2021; Prakas et al., 2021; Rudaitytė-Lukošienė et al., 2022). The differentiation of species can be done through cysts' morphological studies but with certain limitations since the morphological features of some species are similar (Dubey et al., 2016). In recent years, studies based on molecular biology (PCR and sequencing) have allowed the identification and differentiation of several *Sarcocystis* spp*.*, being more practical, precise, and reliable than traditional morphological methods (Gazzonis et al., 2019; Gjerde, 2013; Huang et al., 2019; Moré et al., 2013; Rubiola et al., 2020; Rosenthal, 2021). In this respect, genetic markers such as *18S rRNA* (18S ribosomal RNA), *coxI* (mitochondrial cytochrome *c* oxidase subunit I), and *ITS1* (internal transcribed spacer 1) have allowed reliable diagnosis and the identification of phylogenetic relationships among *Sarcocystis* species (Gazzonis et al., 2019; Huang et al., 2019; Prakas et al., 2020).

The present study provides the first report and molecular characterisation of a *Sarcocystis* spp. producing cysts in muscles of *L. maximus* in Argentina.

## 2 Materials and methods

### 2.1 Sample collection

Muscle samples (tongue, diaphragm, masseter, heart, and hind limbs) were collected from a total of 53 plains viscacha specimens from three provinces of Argentina: *Buenos Aires* (*n* = 28), *Entre Ríos* (*n* = 12) and *Santiago del Estero* (*n* = 13), between 2017 and 2022 (Table 1). They were transported refrigerated (4 °C) and immediately processed or kept frozen (−20 °C) until processing. Samples of each animal were analysed according to the methodology described by Moré et al. (2011). Briefly, 5–10 g of pooled muscle were grounded in a tissue homogeniser with the addition of 50 ml phosphate-buffered saline (PBS - pH 7.2) and then filtered, using a strainer with gauze, collected in a 50 ml tube, and centrifuged at 600×*g* for 5 min. Approximately 3 ml homogenate aliquots were placed in a Petri dish, diluted with PBS and observed in an inverted microscope at 40× magnification (Nikon, TMZ). An aliquot of each homogenate was collected in 1.5 ml DNase-free microtubes and preserved at −20 °C for molecular studies. Samples containing at least one *Sarcocystis* sp. cyst (complete or a fraction) were considered positive.

**Table 1:** Detail of specimens examined of *Lagostomus maximus* from Argentina and results of light microscopy.

Site	Department	Province	Coordinates	Number of specimens	Collection Date	*Sarcocystis* sp. positive specimens	Samples/Cysts per plate
ECAS^a^	Berazategui	Buenos Aires	34°50′47.88″S, 58°6′16.48″W	13	2017–2018	0	–
Bahía Samborombón	Punta Indio	Buenos Aires	35°16′24.21″S, 57°14′52.42″W	2	2018	1	1/1
Islote de la Gaviota Cangrejera	Bahía Blanca	Buenos Aires	38°49′15.80″S, 62°16′13.40″W	1	2019	0	–
Estancia La Bombilla	Tornquist	Buenos Aires	38°32′40.78″S, 62°34′5.87″W	10	2019	0	–
Daireaux	Daireaux	Buenos Aires	36°35′58.48″S, 61°44′51.26″W	2	2022	0	–
Estancia Palmira de Carpinchorí	Federal	Entre Ríos	30°40′43.06″S, 58°40′40.61″W	12	2018	0	–
Estancia Los Quebrachitos	Aguirre	Santiago del Estero	29°1′46.70″S, 62°50′59.79″W	13	2021	6	4/1-4 2/≥ 4

a ECAS: *Estación de cría de Animales Silvestres, Ministerio de Desarrollo Agrario, Provincia de Buenos Aires, Argentina*.

### 2.2 Molecular analysis

The DNA was extracted from pooled muscle homogenates (only for positive samples at microscopy) and individual cysts using a commercial kit according to the manufacturer's instructions (PuriPrep T-kit, Inbio Highway, Argentina). The lysis step was carried out overnight at 55 °C. Each DNA extraction routine was conducted with a process control (a sample using only the kit solutions). The DNA samples were processed by PCR to three marker fragments: *18S rRNA, coxI* (cysts and muscle homogenates), and *ITS1* (only homogenates). All the amplifications were done in a thermocycler (T18, Ivema, Argentina), using a recombinant *TaqDNA* polymerase (Invitrogen™, Brazil), and each PCR routine included a negative control (extraction process control), a no template control (NTC, ultrapure water), and a positive control (*S. falcatula-*like DNA; Origlia et al., 2022). Fragments of *18S rRNA* of 850 bp were amplified by PCR using *SarcoFext* and *SarcoRext* primers in a final volume of 25 μl using a previously described protocol (Moré et al., 2013). Regarding the *coxI* gene marker, fragments of around 1100 bp were amplified by PCR using *SF1* and *SR5* primers (Gjerde, 2013) in a final volume of 25 μl under the following conditions: initial denaturation at 94 °C for 5 min, 45 cycles of 94 °C for 30 s, 53 °C for 30 s and 72 °C for 90 s, and final extension at 72 °C for 5 min.

Regarding the *ITS1* marker, fragments from about 1200 bp were amplified using *SU1F* and *5.8SR2* primers (Gjerde, 2014) in a final volume of 25 μl under the following conditions: initial denaturation at 94 °C for 5 min, 45 cycles of 94 °C for 30 s, 55 °C for 30 s and 72 °C for 90 s, and final extension at 72 °C for 5 min.

Five μl of each PCR product were examined in 1.5% agarose gel stained with SYBRsafe and observed in a blue light transilluminator (Invitrogen, USA). In cases where the PCRs were negative, new PCRs with 1/10 dilutions of DNA samples were carried out (potential presence of inhibitors).

Amplicons of the *18S rRNA*, *coxI*, and *ITS1* PCR (with an estimated concentration of at least 40 ng/μl) were purified using a commercial kit according to the manufacturer's instructions (Wizard SV clean-up system, Promega). They were submitted for Sanger sequencing to Macrogen Inc., South Korea (http://www.macrogen.com), together with the two primers used for each amplification. Sequences obtained were aligned and analysed using the Geneious software (R9 version) (https://www.geneious.com). The consensus sequences obtained were compared with others reported in GenBank by nucleotide BLAST analysis (http://blast.ncbi.nlm.nih.gov/Blast.cgi). The *18S rRNA* and *coxI* consensus sequences obtained were aligned with other sequences of *Sarcocystis* spp. using small mammals, birds, and ruminants as IH and distance trees were constructed using the Neighbor-Joining method based on the Tamura-Nei genetic distance model, with 1000 bootstraps and using *T. gondii 18S rRNA* (M97703) and *coxI* (JX473257) sequence as the outgroup to root the tree, respectively (Geneious, R9).

## 3 Results

### 3.1 Direct microscopy examination

**Fig. 1:**
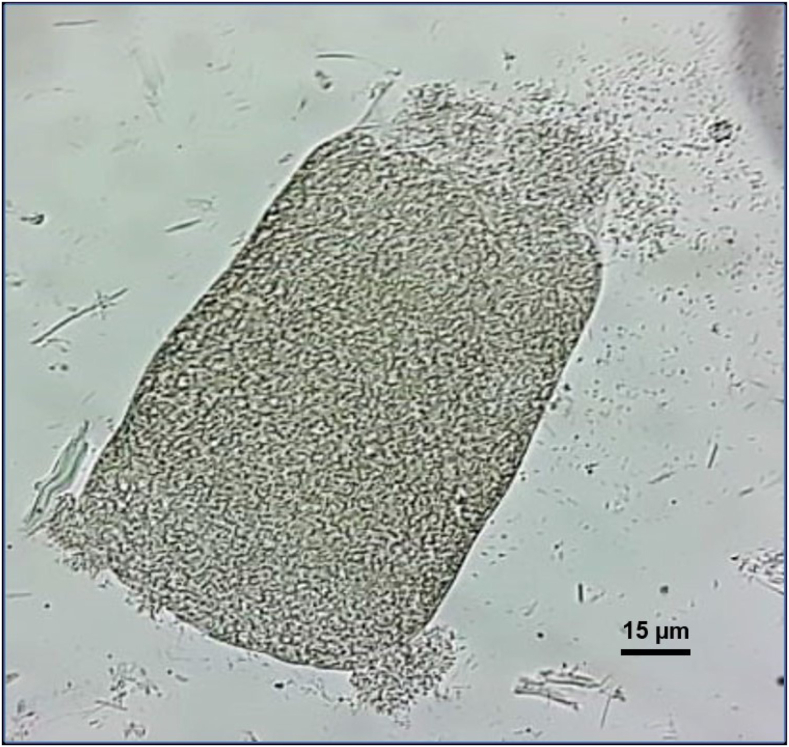
Photograph at inverted microscope at 40× magnification of a sarcocyst portion from a plains viscacha muscle pool.

*Sarcocystis* sp*.* cysts were observed in 7 of 53 (13.2%) plains viscachas. One specimen was from *Bahía Samborombón*, *Buenos Aires* province, and the other six were from *Estancia Los Quebrachitos*, *Santiago del Estero* province. Samples showed a low number of sarcocysts (1–4 per analysed aliquot), except for two samples from *Santiago del Estero* that showed a moderate number of sarcocysts (≥4 per analysed aliquot) (Table 1). All sarcocysts were microscopic, septate, and thin-walled, appearing to belong to one morphological type. Most positive samples showed cyst portions of 150–200 μm (Fig. 1), and the few complete cysts measured up to 500 μm. Bradyzoites presented a banana-shaped structure.

### 3.2 Molecular analysis

Out of seven positive samples by optical microscopy analysis, five were positive by PCR for *18S rRNA* and *ITS1,* and six for *cox1,* all of them by using muscles homogenate DNA. Four samples gave PCR products in the three markers suitable for sequencing, while another was sequenced only in *ITS1* and *cox1* markers. All the PCR samples were from *Santiago del Estero* province. The sequences obtained were deposited in the GenBank under accession numbers: **OP937321**, **OP937322**, **OP937323**, **OP937324**, **OP937325** for the *18S rRNA,* and **OP936996**, **OP936997**, **OP936998**, **OP936999** and **OP937000** for *coxI*.

Seven samples were processed for *18S rRNA* PCR from individualised cysts; only one was positive and with appropriate PCR products for sequencing. All the negative samples were repeated at 1/10 dilution and resulted negative again. Five consensus sequences (four from homogenates and one from a single cyst), ranging from 847 to 877 bp were obtained for *18S rRNA*. The chromatograms from the homogenates showed some double picks and a background signal. The sequences were 99.88–100% similar (only one single base difference), except for the sequence from the individual cyst, which shows a 97.11% homology with the other four. All the sequences showed only 96.89–97.37% identity with reported sequences of *S. singaporensis* (KY513624), *S. zuoi* (KU341120 and JQ029113), *S. nesbitti* (HF544323), *S. attenuati* (MZ826982), *S. masoni* (KU527108) and *S. tarandi* (GQ250970).

For *coxI*, five consensus sequences (each from a muscle homogenate sample), ranging from 989 to 1014 bp, were obtained and showed a 100% identity. The single cyst DNA resulted weak positive and was not sequenced. The sequences showed an identity of 99.41–99.48% with *S. canis* (KX721497) from the brain of a dolphin (*Tursiops aduncus)*, *Sarcocystis* sp. from micromammals in Spain (MT418689), and *Sarcocystis* sp. ex *Pantherophis alleghaniensis* (KU891603).

**Fig. 2:**
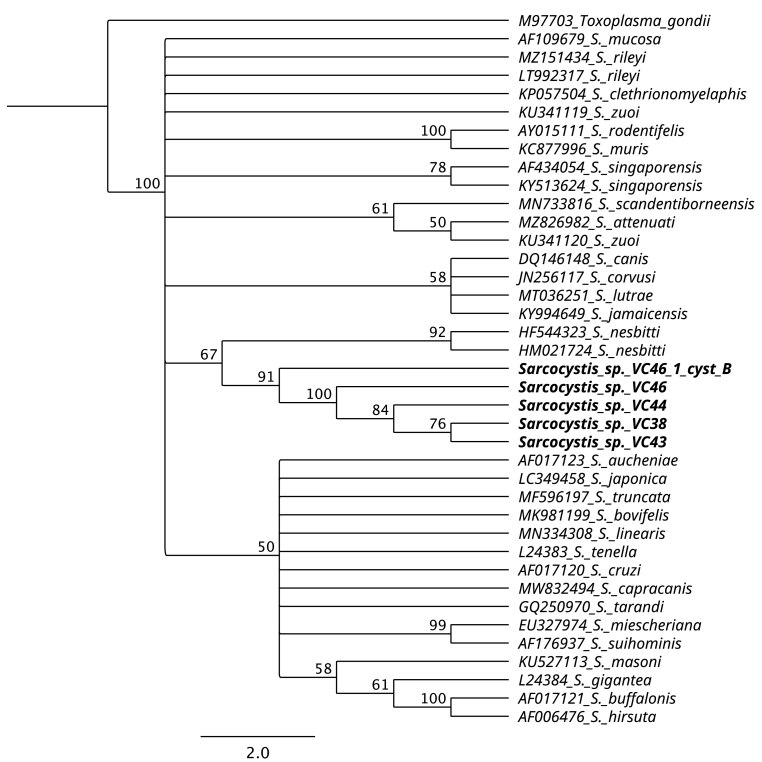
Phylogenetic tree of *18S rRNA* using the Neighbor-Joining method. Sequences obtained from muscles samples and individual cysts of *Lagostomus maximus* (VC46_1_cyst_B, VC46, VC44, VC38, and VC43) form a separate clade of other *Sarcocystis* species and *S. nesbitti* sequences formed a sister branch.

**Fig. 3:**
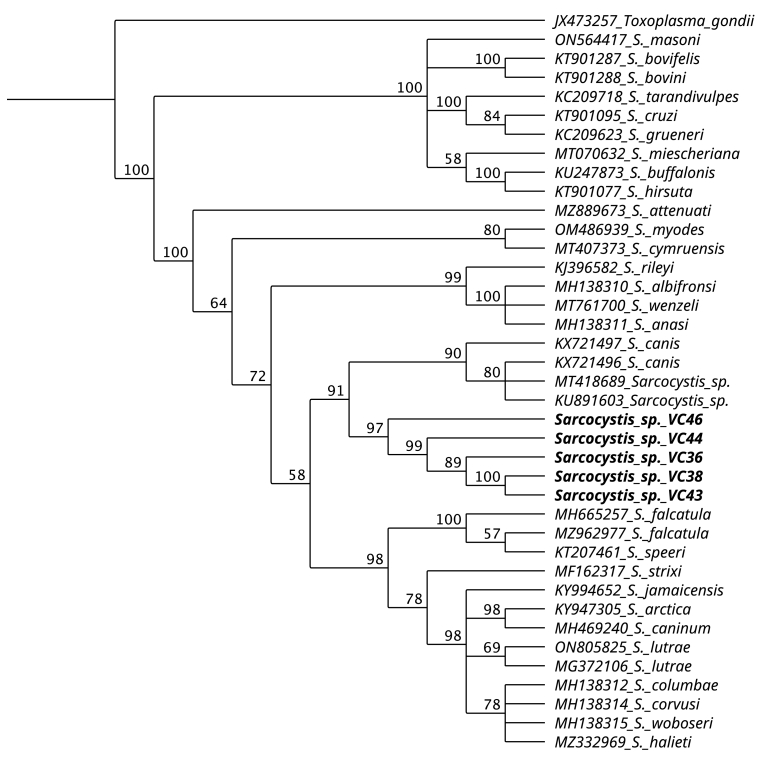
Phylogenetic tree of *coxI* using the Neighbor-Joining method. Sequences obtained from muscles samples of *Lagostomus maximus* (VC46, VC44, VC36, VC38, and VC43) form a separate clade of other *Sarcocystis* species with a sister clade formed by *S. canis* and *Sarcocystis* sp. sequences.

Sequences of *ITS1* were shorter than expected, of low quality (with several double peaks), and consensus sequences could not be constructed. The distance tree constructed with 39 *18S rRNA* sequences showed that all *Sarcocystis* spp. sequences from this study were grouped in a branch with a consensus support of 91%. The closest species was *S. nesbitti,* forming a sister group (Fig. 2). The distance tree constructed with 39 *coxI* sequences positioned the sequences of this study in a well-supported group separated from the rest of the *Sarcocystis* species. In a sister clade were positioned sequences of *Sarcocystis* sp. from *Mus spretus* in Spain and Eastern rat snake in the USA, as well as *S. canis* sequences, with a support of 91% (Fig. 3).

## 4 Discussion

In the present study, the presence of *Sarcocystis* cysts in *L. maximus* is reported for the first time. Furthermore, the identified species were characterised molecularly by sequencing two genetic fragments. So far, there are only three reports of detecting Sarcocystidae protozoan cysts in rodents of the Chinchillidae family (Rakich et al., 1992; Dubey et al., 2016) and only one in *L. maximus* (Cwirenbaum et al., 2021). The cysts described in *L. maximus* from a previous study in Argentina probably belong to *Besnoitia* sp. considering the type (without clear septa) and localisation (Cwirenbaum et al., 2021). The present study detected septated and thin-walled muscular cysts in 7/53 animals. The cysts observed resembled a *Sarcocystis* sp. However, further studies will allow accurate morphological description, particularly the TEM cyst-wall type as proposed for new species (Dubey et al., 2016). Regarding distribution, only one sample from 28 obtained in *Buenos Aires* province resulted positive, while 6/13 from *Santiago del Estero* were positive, and two showed a moderate number of cysts. Despite the low number of samples, it is possible to suggest that the potential DH of the observed *Sarcocystis* spp. should be a frequent predator of plains viscachas with a higher prevalence in *Santiago del Estero*.

Almost all the consensus sequences obtained in the present study from muscles homogenate DNA showed a high identity, suggesting the presence of a single species. However, the observation of some double picks and the background signal in these sequences could suggest that DNA of more than one species was amplified. Despite of this, we obtained good consensus sequences, probably because one of them is overrepresented in the mix. In addition, the *18S rRNA* sequence obtained from a single cyst showed a low identity with the other four sequences. Altogether, indicates that at least two *Sarcocystis* spp. species were present in plains viscacha muscles. Nevertheless, all the obtained *18S rRNA* sequences showed only around 96.9–97.4% identity with other *Sarcocystis* spp. sequences. These homology levels are lower than the recognized for *a Sarcocystis* sp. specific identification (Gjerde, 2013; Moré et al., 2013). Therefore, it is possible to suggest that the obtained *18S rRNA* sequences from plains viscachas correspond to a two closely related and unreported species. On the other hand, the BLAST comparison of *coxI* sequences obtained in this study shows a high similarity with *S. canis* (99.48%) and a *Sarcocystis* sp. identified in *Mus spretus* muscles in Spain and faecal samples from Eastern rat snake (*Pantherophis alleghaniensis*) in the USA. Infections attributable to *S. canis* were reported in a wide range of hosts, including *Canis lupus familiaris* (initial report), *Ursus americanus*, *Ursus maritimus*, *Zalophus californianus*, *Equus caballus*, *Eumetopias jubatus*, *Monachus schauinslandi, Stenella coeruleoalba* and *Chinchilla* sp. (Rakich et al., 1992; Dubey et al., 2016). This species has a unique multiple host pattern, similar to *S. neurona* (Dubey et al., 2016). It is important to remark, that the primers used for *cox1* are one universal (SF1) for *Sarcocystis* spp. and the reverse (SR5) is restricted to a group of species (Gjerde 2013). Therefore, we obtain good quality chromatograms (and without doble picks or background) for *cox1* sequences probably by “selecting” one of the species present in muscles with the reverse primer.

Despite obtaining proper amplicons with validated primers for *Sarcocystis* spp. *ITS1,* the sequences were shorter than expected, and no consensus sequence was reached. This could be due to the DNA amplification of two or more different species (as previously mentioned for the *18S rRNA*) as well as the high repeatability and variability reported for this marker, which could lead to reading problems in the Sanger sequencing (Gjerde, 2014). Future studies should be conducted using single cyst PCR to reach unique amplicons, as previously performed in other *Sarcocystis* spp. from rodent muscles (Prakas et al., 2019; Rudaitytė-Lukošienė et al., 2022).

The analysis of genetic distance trees with *18s rRNA* and *coxI* sequences shows that all *Sarcocystis* sequences obtained in this study were grouped with high support and having as sister clade sequences from *S. nesbitti,* and *S. canis* and *Sarcocystis* sp. from micromammals and snake, respectively. In both trees, our sequences were separated from the other *Sarcocystis* spp. used in the alignments. However, considering that there is no higher homology at the *18S rRNA* marker, the positioning in the trees could vary if sequences with higher degrees of similarity were available. Since *S. nesbitti* and *Sarcocystis* sp. from snake faeces show a closer genetic distance with *Sarcocystis* species studied here, snakes could be suggested to be the DH of some of them (Fayer et al., 2015; Dubey et al., 2016). Nevertheless, the close relation of *cox1* sequences with those of *S. canis*, suggests that plain vizcachas could also being infected with this specie, as previously reported for *Chinchilla* sp. (Rakich et al., 1992). *Sarcocystis canis* is a species with multiple IH and uncertain DH, could indicate that hosts other than *L. maximus* may be infected, and even canids could be DH. Canids and snakes are found in sympatry with *L. maximus* and are considered possible natural predators of this rodent. It has also been reported that *Boa constrictor* could be a natural predator of the plains viscacha, and its distribution area includes *Santiago del Estero* province, where the most significant number of plains viscacha with *Sarcocystis* spp. cysts was found and also all the sequenced samples came from (Jackson et al., 1996; Arzamendia et al., 2021). Likewise, in Argentina, there are four species of canids whose distributions coincide to a greater or lesser extent with that of the plains viscachas in which *Sarcocystis* cysts were found: *Canis lupus familiaris, Cerdocyon thous, Chrysocyon brachyurus,* and *Lycalopex gymnocercus* (Cirignoli et al., 2019a, 2019b; Lartigau et al., 2019; Luengos Vidal et al., 2019)*.* Nevertheless, further studies are needed to identify the DH of the *Sarcocystis* spp. producing sarcocysts in plains viscacha and clarify its IH range and zoonotic potential. Notably, plains viscacha is frequently consumed by humans, and the presence of *Sarcocystis* spp. could be underestimated.

## 5 Conclusions

In this paper, the molecular characterisation of a *Sarcocystis* species was carried out from *L. maximus* muscles*,* a host that had not been previously recorded for this genus. Given the host specificity that *Sarcocystis* spp. can present and the lower identity with other reported sequences for *18S rRNA* marker, the species in plains viscacha from Argentina are potentially new species. In addition, regarding the *coxI* sequences obtained here, viscachas could be HI for a *Sarcocystis canis*-like species. Regarding nomenclature, we considered the species to be named as new once further morphological descriptions become available. In addition, further studies are necessary to elucidate definitive hosts and the zoonotic potential of the species producing sarcocysts in plains viscacha.

## Ethical statement

The research was conducted according to Argentine laws. Sample collection was carried out during fieldwork under official permission granted by the *Dirección de Minería, Medio Ambiente y Recursos Naturales,* province of *Entre Ríos*, the *Dirección de Flora y Fauna*, province of *Buenos Aires,* and the *Dirección General de Bosques y Fauna*, province of *Santiago del Estero*, and by the recommendations of the Guidelines for the capture, handling and care of mammals as approved by the American Society of Mammalogists (Animal Care and Use Committee 1998). No endangered species were involved in this study.

## Funding

This work was partially supported by *Agencia Nacional de Promoción Científica y Tecnológica* (PICT, 2019-370) and 10.13039/501100003947Universidad Nacional de La Plata (No. 861), Argentina.

## Author's contributions

VC and AMA collected the host specimens. VC, EH, and GM examined the muscle samples. GM did the molecular analysis. All authors discussed the result and contributed to the final manuscript.

## Declaration of competing interest

The authors declare that they have no known competing financial interests or personal relationships that could have appeared to influence the work reported in this paper.
